# On-chip modulation for rotating sensing of gyroscope based on ring resonator coupled with Mach-Zehnder interferometer

**DOI:** 10.1038/srep19024

**Published:** 2016-01-22

**Authors:** Hao Zhang, Jiayang Chen, Junjie Jin, Jian Lin, Long Zhao, Zhuanfang Bi, Anping Huang, Zhisong Xiao

**Affiliations:** 1 Key Laboratory of Micro-nano Measurement-Manipulation and Physics (Ministry of Education), School of Physics and Nuclear Energy Engineering, Beihang University, Beijing 100191, China

## Abstract

An improving structure for resonance optical gyro inserting a Mach-Zehnder Interferomete (MZI) into coupler region between ring resonator and straight waveguide was proposed. The different reference phase shift parameters in the MZI arms are tunable by thermo-optic effect and can be optimized at every rotation angular rate point without additional phase bias. Four optimum paths are formed to make the gyroscope to work always at the highest sensitivity.

Chip-scale optical inertial rotation sensors^1^,^2^,^3^,^4^,^5^,^6^ based on Sagnac effect^7^ have distinct advantages in aspects of physical size, weight, power consumption, cost, compatibility with technology for producing microelectronics, and have the most potential to become the next generation optical gyroscopes after ring laser gyro (RLG) and fiber optic gyro (FOG). Nevertheless, on-chip optical gyroscope with tiny area is bound to accumulate less phase shift and less sensitive than RLG and FOG. In order to make on-chip optical gyroscope more sensitive, various coupled-cavities structures such as coupled resonator optical waveguides (CROWs) and side-coupled integrated spaced sequence of resonators (SCISSOR) were proposed^8^,^9^,^10^ and slow-light effect were introduced into those structures. However, loss ultimately limits the achievable sensitivity in CROW structure. Physically, slow light is a resultant property of the highly dispersive structure and has no direct link with the enhancement of gyro’s sensitivity^9^. It is beneficial to introduce enhanced effect to increase accumulated frequency or phase shift, such as fast light^11^,^12^,^13^,^14^,^15^ or nonlinear Kerr effect^16^,^17^. Another effective way is utilizing ultralow propagation loss of optical waveguide^18^ or introducing active gain into waveguide for compensating loss^19^,^20^,^21^. In addition to enhanced effect and ultralow propagation loss, parameter optimization is an essential part to make the chip size gyroscope work at the most sensitive point^22^. All gyroscopes must be optimized with respect to their experimentally adjustable parameters. In the case of resonant gyroscopes, this means that the sensitivity must be calculated using optimum values for coupling coefficients and phase biases^23^,^24^,^25^. While, the parameters of on-chip rotation sensors are all influenced by processing technology and cannot be changed after fabricating. That is to say, the gyroscope is only designed by static optimization and not dynamic optimization.

An external phase modulator always provides necessary phase bias in conventional interferometric fiber optic gyro (IFOG). This modulator is usually made by using Piezoelectric ceramic (PZT) or electro-optic crystal materials lithium niobate (LiNbO_3_) and phase modulation is produced by driven voltage. Due to fiber winding on Piezoelectric ceramic (PZT), PZT phase modulator is not suitable for integrating on a chip, in addition, the frequency using PZT phase modulation is low which is not satisfied the requirement of feedback control components. On the other hand, Y-branch waveguide made by LiNbO_3_ can produce high modulation frequency and integrate beam splitter, polarizer and modulator on a chip. However, LiNbO_3_ phase modulators were adopted to realize the frequency modulation, which made it incompatible with silicon process technology and standard CMOS electronics, and thus it is harder to integrate into complete systems on a chip. LiNbO_3_ modulator is still high cost with respect to silicon optical modulator. Silicon has the higher refractive index than LiNbO_3_, which is advantaged to size reduction. To the best our knowledge, LiNbO_3_ waveguide propagation loss (0.05 dB/cm^26^) is still higher than silicon based waveguide (0.01 dB/cm^27^) for the transmission wavelength of 1550–1580 nm.

Silicon optical modulator is typical integrating modulator, which could have been realized in both monolithic and hybrid forms. To the present, silicon-based modulators which operate via carrier depletion have been demonstrated at data rates up to 50 Gb/s^28^. Applying an electric field to a material may change the refractive indices (real and imaginary). Unfortunately, the centrosymmetric crystal structure of silicon dose not exhibit the Pockels effect (a linear electro-optical effect) and Kerr effect and Franz–Keldish effect are weak at 1.55 μm^29^. Thus making electro-optic silicon modulators is difficult to realize^30^. Alternative methods are required to achieve modulation in silicon. One option is plasma dispersion effect, in which the concentration of free charges in silicon contributes to the loss via absorption^31^. Generally, in electrorefraction modulators, a balance should be struck between electrorefraction and eletroabsorption^32^. The effect of free carrier losses due to current injection is judged by observing the change in Q-factor during tuning, where any losses will lower the Q-factor. But there is not any change in Q-factor for thermo-optic effect^33^, whereas electro-optic modulators have large insertion loss usually more than 4 dB^34^. And the other practical option is thermal modulation owing to the large thermo-optic coefficient (TO) of silicon^35^,^36^,^37^,^38^. Early in 2000 years K. Suzuki and K. Hotate^39^ had proposed and experimented the countermeasure for Backscattering induced noise via Thermo-optic phase modulation in a compact micro-optic gyro. Moreover, the modulator speed was increased via the application of a thermal bias holding the modulator at a higher average temperature with respect to the substrate heat sink^40^. In 2010, Morichetti. F *et al.* showed that resonator optical waveguide (CROW) delay lines fabricated on a silicon on insulator (SOI) platform at 100 Gbit/s. Heaters for SOI technology reveal to be more than 120 times faster than their counterpart in silica technology^41^. Though thermal modulation is comparatively low speed for high frequencies required by telecommunications applications, for sensing applications only relative moderate modulation speeds are need.

In this paper, an improving resonance structure for gyroscope was proposed, which inserts a Mach-Zehnder Interferometer (MZI) as a coupler between ring resonator and straight waveguide and each arm is introduced a different reference phase shift ΔΦ_1_, ΔΦ_2_, called MZI-coupler resonant waveguide optical gyroscope (MZIC-RWOG) (Fig. 1). When the phase shift in the ring resonator is changed by Sagnac effect, the proposed schematic for integrated optical gyroscope can keep the minimum detectable rotation rate (resolution) value through modulating phase shift using thermo-optic effect in coupled region of MZIC-RWOG structure and do not need to add external phase bias in MZIC-RWOG, which is more suitable for modulating on a chip.

## Results

In this work, we only consider the thermo-optic effects to control reference phase shift ΔΦ_1,2_ in the arms of MZI. As current injected into the MZI electrode, heating changes the waveguide’s refractive index, and introduces a phase shift^35^,^36^:


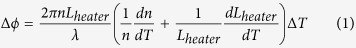


where λ is the wavelength, 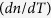
is the thermo-optic coefficient (TO) and 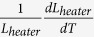 represents thermalexpansion coefficient. ***Δ**T* is the change in the temperature (Kelvin), and *L*_*heater*_ is the heater length. For example, if the *L*_*heater*_ is 500 μm and 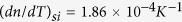
(a typical value for silicon at λ = 1550 nm^38^), a temperature change of 8.3 K is needed to achieve a π-phase shift. The sign and magnitude of the TO coefficient are primarily determined by the material density and polarizability. The change of TO coefficient is due to the difference of the propagation constant and optical confinement, an analytical expression for description of channel waveguides TO coefficient is^42^


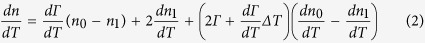


where n_0_ is the channel waveguide refractive index and n_1_ is the cladding refractive index and Γ is the confinement factor which defines the fraction of optical mode inside the core region. If 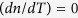, that means to achieve temperature-insensitivity in the effective index of a waveguide device by balancing the overall TO coefficient using with a negative TO coefficient of cladding to compensating positive TO coefficient of the core. However, our aims are temperature-sensitivity and controlling the phase shift through thermo-optic effect. We define the slope *S (K*^−1^) between phase shift and temperature change is 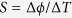.

Figure 2 show the slope *S (K*^−1^) versus the material TO coefficients of core with different confinement factors Γ = 50%, 70%, 90% when the material TO coefficients of cladding is set to −0.0003 K^−1^, 0 K^−1^ and 0.0003 K^−1^. Here confinement factor Γ depend only on the n_0,1_ and waveguide cross section geometry at room temperature. Thermal expansion coefficient is ignored since much smaller than TO coefficient in silicon. Moreover, a smaller confinement factor Γ can get the larger *S (K*^−1^) if material TO coefficients of cladding is negative (see Fig. 2(a)) and a larger confinement factor Γ can get the larger *S (K*^−1^) if material TO coefficients of cladding is positive (see Fig. 2(c)).

**Figure 1:**
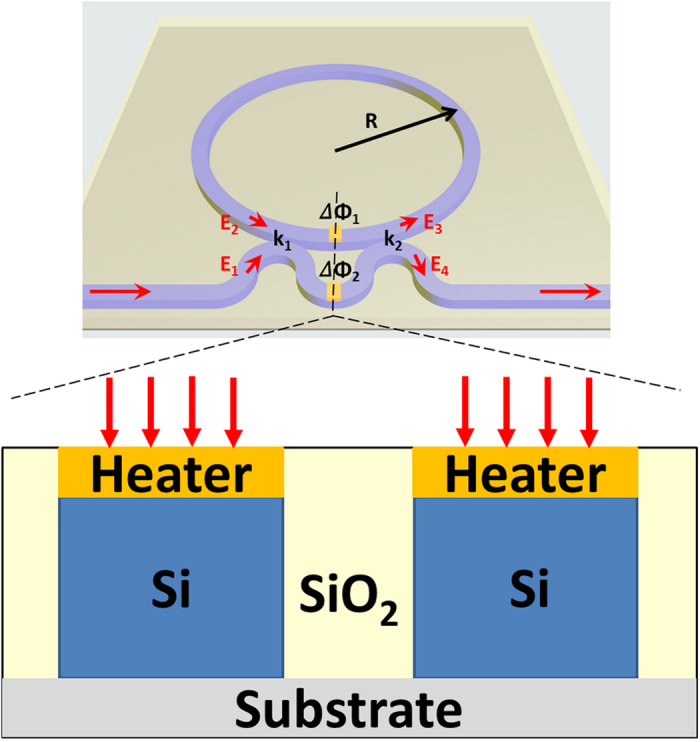
MZI-coupler resonant waveguide optical gyroscope (MZIC-RWOG).

**Figure 2:**
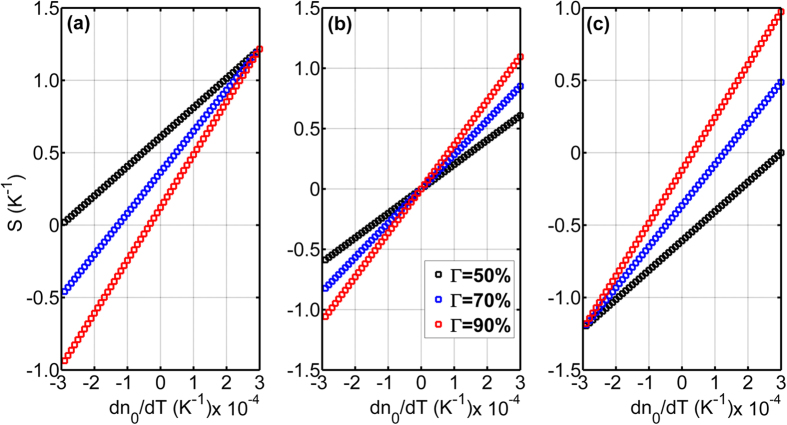
The slope S between phase shift and temperature change versus the material TO coefficients of core for different confinement factor Γ = 50%, 70%, 90% when λ = 1550 nm, L_heater_ is 500 μm. The material TO coefficients of cladding are (**a**) −0.0003 K^−1^, (**b**) 0 K^−1^ and (**c**) 0.0003 K^−1^.

Next, the transmission characteristic of MZIC-RWOG in Fig. 1 can be obtained by

using transfer matrix approach^43^,


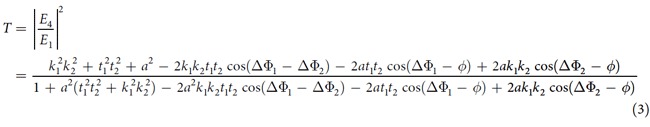


where *k*_*1*_, *k*_*2*_ are the coupling coefficient and *t*_*1*_, *t*_*2*_ are the through coupling coefficient of directional couplers respectively. When the coupling loss is neglected, 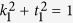 and 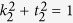 are satified. The one round-trip amplitude propagation attenuation is 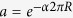, in which α is the propagation loss with the units of m^−1^, and ϕ is the phase shift a light experiences one round-trip, which is composed by two terms:


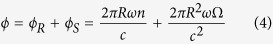


where w, n, c and Ω is the angular frequency of the light, effective index of the ring, the speed of light in vacuum and rotation angular rate, respectively. The second term ϕ_s_ in equation (4) is the phase shift induced by the Sagnac effect when the sensor rotate about an axis perpendicular to the plane of optical resonator at an angular velocity Ω.

By comparing the transfer function of MZIC-RWOG with all-pass resonant waveguide optical gyroscope (RWOG), which is composed by one straight waveguide and a single ring resonator, the effective through coupling coefficient *t*_*eff*_ can be found


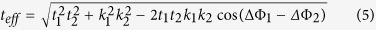


According to^22^,^44^,^45^,^46^, the resolution 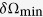
(which is defined as the minimum detectable angular rate) is expressed if the resonator is circular:


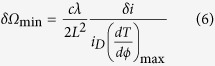


The amplitude noise term 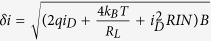 and *i*_*D*_ are the standard deviation of the photocurrent and the maximum photodiode current (specific parameters are listed in Table 1).

## Discussion

In order to indicate the enhancement effect on sensitivity of MZIC-RWOG and compare the sensitivity of MZIC-RWOG with RWOG, we must firstly optimize the parameters of RWOG. The necessary global optimizing parameters include the area of ring resonator A, the coupled coefficient k between ring resonator and straight waveguide, the round-trip propagation loss α if the coupled loss is neglected and the phase shift ϕ which ensures the gyroscope obtain maximum sensitivity when rotation rate is null (so-called bias phase shift ϕ_bias_). According to transfer matrix approach, the analytical expression of the all-pass structure RWOG for the minimum detectable angular rate 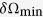 can be calculated which is a function of A, k, α and ϕ. Although it is possible to optimize 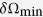 using the exact formula, numerical calculation for obtaining minimum value of 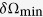 is more convenient. More specifically, we can scan parameters (A, k, α, ϕ) and calculate every value of resolution 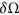 in a setting range, then find out both the minimum value δΩ_min_ and optimizing parameters pair (A_opt_, k_opt_, α_opt_, ϕ_bias_). For the optimizing parameters pair (A_opt_, k_opt_, α_opt_, ϕ_bias_), except ϕ_bias_ the other three parameters are all determinated by processing technology and cannot be changed after fabricating.

**Figure 3:**
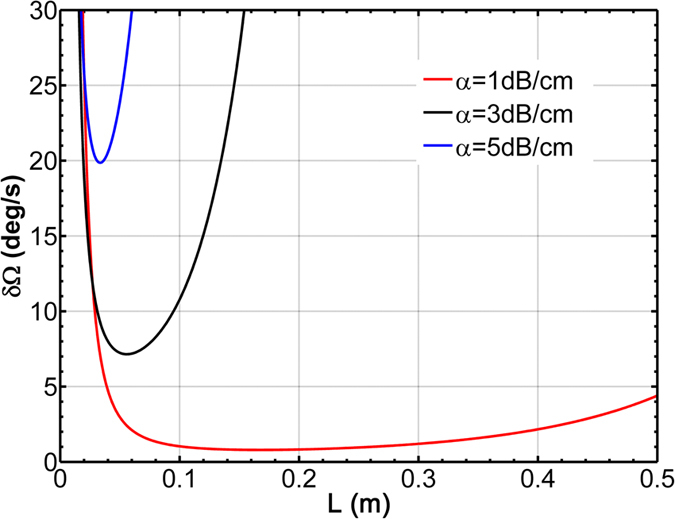
Rotation rate resolution versus resonator perimeter for the RWOG. The parameters ϕ and k are all fixed at optimum value (C_IL_ = 1).

For the level of current processing technology the propagation loss of silicon based waveguide is about 1–5 dB/cm and every value of propagation loss is according to an optimum perimeter for a RWOG. All the parameter pair (*L, k, ϕ*) are scanned to find out the minimum value of 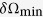
after setting a fixing value for propagation loss. From Fig. 3 we can see that there is always an optimum *L* corresponding to 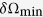 for every loss value and the optimum parameters are shown as Table 2.

**Figure 4:**
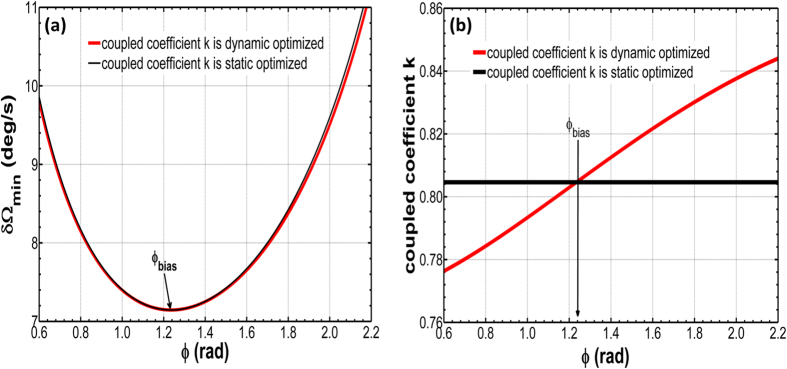
(**a**) Rotation rate resolution versus phase shift in the resonator when k is fixed at static optimization point 0.8046 (black line) Rotation rate resolution versus phase shift in the resonator when k is tunable (red line) (**b**) the optimized path of k is one-to-one corresponding to (**a**). All the case are assumed α = 3 dB/cm, L_opt_ = 0.0554 m, ϕ_bias_ = 1.2353.

Note that the optimizing parameters pair (A_opt_, k_opt_, α_opt_, ϕ_bias_) only ensure maximum sensitivity at zero rotation-rate point since the parameters have been fixed after fabrication, thus the parameters (A_opt_, k_opt_, α_opt_, ϕ_bias_) pair is not optimal when the gyroscope is rotating. That is to say, the gyroscope is only designed by static optimization and not dynamic optimization because the coupled coefficient k cannot be controlled after fabrication. If it assumes that coupled coefficient *k* is tunable for every Sagnac phase shift *ϕ*_*s*_ caused by rotation angular rate *Ω*, then we can find out a path of dynamic optimization of *k* to obtain minimum *Ω*. In Fig. 4(a), it shows that the two situations: *k* is fixed at static optimization point 0.8046 (black line) and k is tunable (red line). Although the resolution 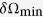 is the same at *ϕ*_*bias*_ for the two situations, there is small enhancement for 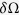 with the Sagnac phase shift *ϕ*_*s*_ increasing when k is tunable and the dynamic optimized path for k is shown in the Fig. 4(b) which is one-to-one corresponding to Fig. 4(a). From Fig. 4(b) the value of coupled coefficient k ranges from 0.7763 to 0.8440 which is corresponding to a range of gap between ring resonator and straight waveguide.

Next we will analyze the structure of MZI-coupler resonant waveguide optical gyroscope (MZIC-RWOG) showed in Fig. 1 and show that MZIC-RWOG has higher sensitivity than RWOG via dynamic optimization. For a MZIC-RWOG there is need to optimize parameters pair (*L*_*m*_*, k*_*m*_*, α*_*m*_*, ϕ*_*m*_, ΔΦ_1_, ΔΦ_2_), and the subscript m here indicates the parameters of MZIC-RWOG. Note that we assume *k*_*m*_ = *k*_*1*_ = *k*_*2*_ here, because *k*_*1*_ *, k*_*2*_ are symmetrical in the equation (3), so the optimum value of *k*_*1*_ and *k*_*2*_ must be the same. In addition, the propagation loss *α*_*m*_ is assumed to be 3 dB/cm and *C*_*IL*_ = 1 from now on, unless stated otherwise.

As in the case of RWOG, all the parameters pairs (*L*_*m*_*, k*_*m*_*, α*_*m*_*, ϕ*_*m*_, ΔΦ_1_, ΔΦ_2_) could be scanned and find out optimum parameters pairs for minimum resolution 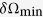. It is worth noting that there is not only one pair *Φ* (*ϕ*_*m*_, ΔΦ_1_, ΔΦ_2_) when *L*_*m*_ and *k*_*m*_ are fixed at optimum values. That is to say, there are many pairs (*L*_*m-opt*_*, k*_*m-opt*_, Φ(*ϕ*_*m*_, ΔΦ_1_, ΔΦ_2_)) for minimum resolution 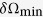. Then if bias phase shift *ϕ*_*m-bias*_ is set to zero, one of optimum parameters pairs could be found (*L*_*m-opt*_ = 0.0554, *k*_*m-opt*_ = 0.461, *ϕ*_*m-bias*_ = 0, ΔΦ_1-opt_ = 1.3635, ΔΦ_2-opt_ = 1.7279), which we will discuss specifically later in this paper. Furthermore, the minimum resolution of MZIC-RWOG is the same with RWOG (0.1247 deg/s) when they have the same perimeter. It is well understood that the optimum perimeters (*L*_*opt*_*, L*_*m-opt*_) are equal for both MZIC-RWOG and RWOG due to the same propagation loss, so the Sagnac effect is not enhanced in a MZIC-RWOG. Indeed, the MZIC-RWOG discussed in this paper dose not enhance the absolute sensitivity for gyro, because the Sagnac effect is relate to the enclosed area which is not changed for MZIC-RWOG. The optimum perimeter *L*_*m-opt*_ and coupled coefficient *k*_*m-opt*_ of MZIC-RWOG have been obtained and these two parameters cannot be changed once the resonator on the chip is fabricated. Hence the only tunable parameter is *Φ*(*ϕ*_*m*_, ΔΦ_1_, ΔΦ_2_) after fabricated. According to equation (4), *ϕ*_*m*_ is composed by bias phase shift *ϕ*_*m-bias*_ and Sagnac phase shift *ϕ*_*m-Sagnac*_ (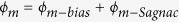). Once bias phase shift *ϕ*_*m-bias*_ is determined and to be a constant, *ϕ*_*m*_ is only increased as the Sagnac phase shift *ϕ*_*m-Sagnac*_. Along with an increasing Sagnac phase shift *ϕ*_*m-Sagnac*_, thus, we have three methods to optimize the resolution 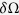: a) ΔΦ_1_ is tunable, ΔΦ_2_ is fixed at optimum value; b) ΔΦ_1_ is fixed at optimum value, ΔΦ_2_ is tunable; c) ΔΦ_1_ and ΔΦ_2_ are both tunable.

**Figure 5:**
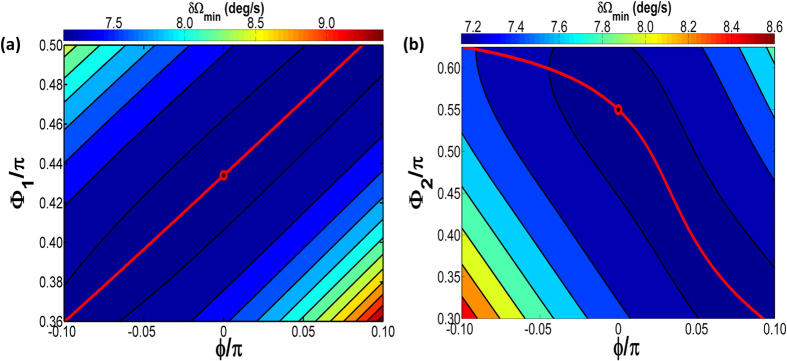
(**a**) The optimum path of Δ*Φ*_*1*_ when Δ*Φ*_*2*_ is fixed at optimum value 1.7279. (**b**) The optimum path of Δ*Φ*_*2*_ when Δ*Φ*_*1*_ is fixed at optimum value 1.3635. All the case are assumed α = 3 dB/cm, L_m-opt_ = 0.0554 m, k_m-opt_ = 0.461.

In the following sections, we will discuss specifically the three methods and the control effect for rotation rate resolution 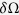 respectively. The red line in Fig. 5(a) shows the optimum dynamic path of ΔΦ_1_ versus the Sagnac phase shift *ϕ*_*m-Sagnac*_ when ΔΦ_2_ is fixed at optimum value ΔΦ_2-opt_. In the middle of the contour the optimum path of ΔΦ_1_ goes through the black point, which is the global optimized point (ΔΦ_1-opt_, ΔΦ_2-opt_) when bias phase shift *ϕ*_*m-bias*_ is set to zero. The maximum optimized range for rotation rate resolution is approximately 2 deg/s resulted from the colorbar in Fig. 5(a). Meanwhile, the red line in Fig. 5(b) shows the optimum dynamic path of ΔΦ_2_ versus the Sagnac phase shift *ϕ*_*m-Sagnac*_ when ΔΦ_1_ is fixed at optimum value ΔΦ_1-opt_. The optimum path of ΔΦ_2_ goes through the black point, which is the global optimized point (ΔΦ_1-opt_, ΔΦ_2-opt_) when bias phase shift *ϕ*_*m-bias*_ is set to zero. The maximum optimized range for rotation rate resolution is approximately 1.5 deg/s resulted from the colorbar in Fig. 5(b). The preliminary result is, hence, that optimum trajectory tracking control for ΔΦ_1_ can obtain higher sensitivity than ΔΦ_2_, and the tuning range is less 1.28 times than tuning ΔΦ_2_.

**Figure 6:**
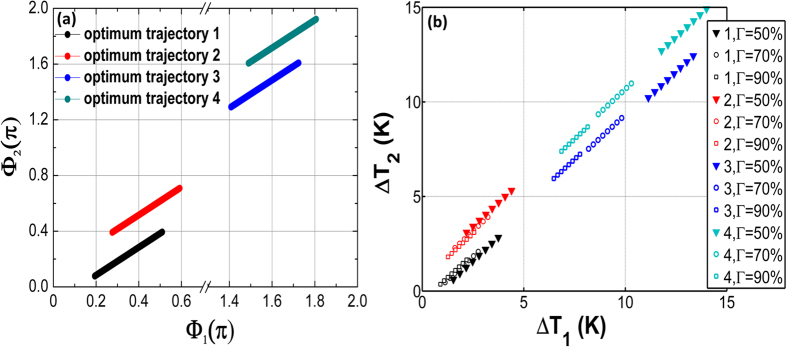
(**a**) The four optimum trajectory lines through tuning Δ*Φ*_*1*_ and Δ*Φ*_*2*_ together when α = 3 dB/cm, L_m-opt_ = 0.0554 m, k_m-opt_ = 0.461. (**b**) The change in the temperature (Δ*T*_*1*_ − Δ*T*_*2*_) is corresponding to (Δ*Φ*_*1*_ −Δ *Φ*_*2*_) in (**a**) with the different confinement coefficient Γ when *L*_*heater*_ = 500 μm, 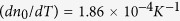, 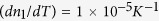 λ = 1550 nm.

By optimizing parameters Φ_1_ and Φ_2_ from 0 to 2π, there are four optimum trajectory lines to make the sensitivity keeping the minimum value with the change of ϕ (Fig. 6(a)). That means when the phase shift *ϕ* is fixed, there are four extreme points at the Φ_1_-Φ_2_ plane from 0 to 2π. Each of optimum line is corresponding to the phase shift ϕ from −0.5 to 0.5. Figure 6(b) shows the change in the temperature (ΔT_1_ − ΔT_2_) corresponding to (ΔΦ_1_ − ΔΦ_2_) in Fig. 6(a) with the different confinement coefficient Γ. High confinement coefficient Γ needs less change of the temperature.

**Figure 7:**
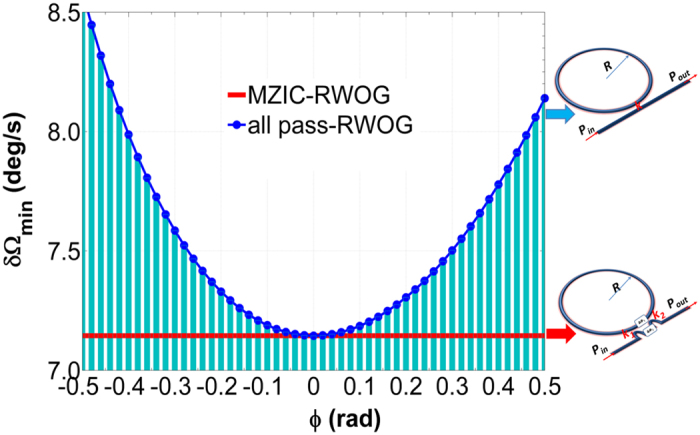
Comparing the resolution of dynamic optimized MZIC-RWOG with the optimized RWOG. All the case are assumed α = 3 dB/cm, L_opt_ = 0.0554 m.

Next, we compared the resolution of dynamic optimized MZIC-RWOG on optimum trajectory with the optimized RWOG in Fig. 7. It is found that the resolution for MZIC-RWOG keeps the minimum value through tuning together Φ_1_ and Φ_2_ follow with the trajectories in Fig. 6 when the phase shift in the ring resonator is changed by Sagnac effect. Dynamic optimization for MZIC-RWOG can play a better performance. This can be explained that we reset the bias phase shift through tuning together ΔΦ_1_ and ΔΦ_2_ all the time, so the resolution keeps the minimum value. Hence, MZIC-RWOG can play a better dynamic performance than RWOG.

Finally, we induce the relation between rotation rate Ω and thermal modulation temperature. It is found that the resolution for MZIC-RWOG keeps the minimum value through tuning together Φ_1_ and Φ_2_ following with the trajectories in Figs 6 and 7 when the Sagnac phase shift *ϕ*_*s*_ in the ring resonator is changed from −0.5 to 0.5. The variation phase difference is 0.88 (rad) for the modulation phase shift Φ_1_ and Φ_2_ from Fig. 6. Since the modulation phase shift Φ_1_ and Φ_2_ are a linear change with the increasing of Sagnac phase shift, the linear relation is


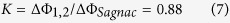


Therefore, we can obtain ΔT as a function of Sagnac phase shift ϕ_s_ via combining equation (1) and (7)


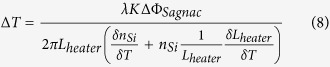


**Figure 8:**
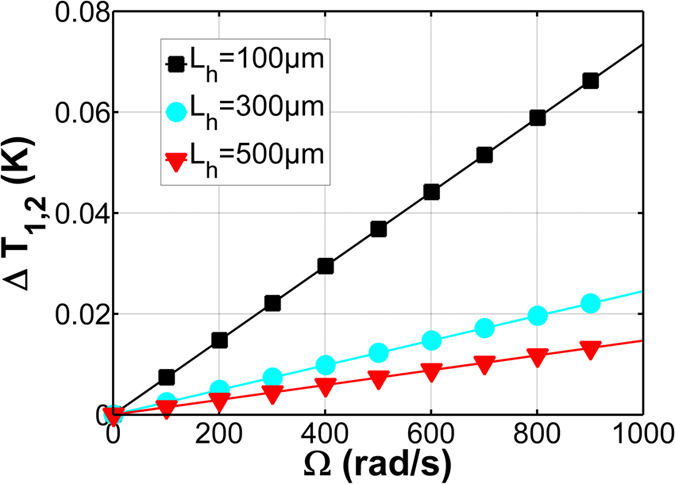
ΔT is as a function of rotation rate Ω with different heater length L_heater_ = 100 μm, 300 μm and 500 μm.

In Fig. 8 shows the modulation temperature is as a function of rotation rate Ω with different heater lengths. The shorter heater length needs larger modulation temperature. The parameters using for simulation are confinement factor Γ is 90%, perimeter of ring resonator L = 0.0554 m, T-O coefficient for silicon is 1.86 × 10^−4^ (K^−1^), thermal expansion coefficemnt is 2.6 × 10^−4^ (K^−1^).

Based on the structure of MZIC-RWOG the optimum dynamic trajectory of reference phase shift ΔΦ_1_ and ΔΦ_2_ is showed when the other parameters were fixed at optimum value. The proposed schematic for integrated optical gyroscope can keep the minimum resolution value through phase-tunable method using thermo-optic effect in coupled region of MZIC-RWOG structure, which is potential for on-chip modulating.

## Methods

An improving resonant gyro called MZIC-RWOG is proposed and the optical field distribution and wave propagation are calculated according to transfer matrix approach. Numerical calculation, which is carried out by Matlab code, for obtaining minimum value of resolution of gyro is used for parameters dynamically optimizing.

## Additional Information

**How to cite this article**: Zhang, H. *et al.* On-chip modulation for rotating sensing of gyroscope based on ring resonator coupled with Mach-Zehnder interferometer. *Sci. Rep.* **6**, 19024; doi: 10.1038/srep19024 (2016).

## Figures and Tables

**Table 1:** List of parameters.

Parameter	Value
Fundamental electric charge	q = 1.6 × 10^−19^
Plank’s constant	h = 6.626 × 10^−34^(J/s)
Optical wave frequency	v = c/λ
Quantum efficiency	η = 0.6813
Input power	P_in_ = 1 mW
Responsivity	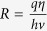
Boltzman’s constant	k_B_ = 1.38 × 10^−23^(J/K)
Photodetector load resistance	R_L_ = 10 Ω
Temperature	T = 298.15 K
Relative intensity noise of the laser	RIN = −160 dB/Hz
Bandwidth	B = 10 Hz

**Table 2:** List of optimum parameters for different propagation loss.

Loss α(dB/cm)	L_opt_ (m)	k_opt_	ϕ (rad)	δΩ_min_ (deg/s)
1	0.1663	0.8047	1.2359	0.0139
3	0.0554	0.8046	1.2353	0.1247
5	0.0333	0.8047	1.2365	0.3464

## References

[b1] Mao H., Ma H. & Jin Z. Polarization maintaining silica waveguide resonator optic gyro using double phase modulation technique. Opt. Express 19, 4632 (2011). 21369295 10.1364/OE.19.004632

[b2] Ma H., Wang W., Ren Y. & Jin Z. Low-Noise Low-Delay Digital Signal Processor for Resonant Micro Optic Gyro. IEEE Photon. Technol. Lett. 25, 198 (2013).

[b3] Feng L., Lei M., Liu H., Zhi Y. & Wang J. Suppresion of back reflection noise in a resonator integrate d optic gyro by hybrid phase-modulation technology. Appl. Opt. 52, 1668 (2013). 23478771 10.1364/AO.52.001668

[b4] Feng L. *et al.* Transmissive resonator optic gyro based on silica waveguide ring resonator. Opt. Express 22, 27565 (2014). 25401903 10.1364/OE.22.027565

[b5] Ciminelli C., Dell’Olio F., Campanella C. E. & Armenise M. N. Photonic technologies for angular velocity sensing. Adv. Opt. Photon. 2, 370–404 (2010).

[b6] Dell’Olio F., Tatoli T., Ciminelli C. & Armenise M. N. Recent advances in miniaturized optical gyroscopes. J. Europ. Opt. Soc. Rap. Public. 9, 14013 (2014).

[b7] Post E. J. Sagnac effect. Rev. Mod. Phys. 39, 475–493 (1967).

[b8] Scheuer J. & Yariv A. Sagnac effect in coupled resonator slow light waveguide structures. Phys. Rev. Lett. 96, 053901 (2006). 16486930 10.1103/PhysRevLett.96.053901

[b9] Yan L., Xiao Z., Guo X. & Huang A. Circle-coupled resonator waveguide with enhanced Sagnac phase-sensitivity for rotation sensing. Appl. Phys. Lett. 95, 141104 (2009).

[b10] Deng S., Xiao Z., Yan L. & Huang A. Optical loss effect on Sagnac sensitivity of circle-coupled resonator structure. Opt. Commun. 290, 76–79 (2013).

[b11] Shahriar M. S. *et al.* Ultrahigh enhancement in absolute and relative rotation sensing using fast and slow light. Phys. Rev. A 75, 053807 (2007).

[b12] Ciminelli C., Campanella C. E., Dell’Olio F. & Armenise M. N. Fast light generation through velocity manipulation in two vertically-stacked ring resonators. Opt. Express 18, 2973–2986 (2010). 20174126 10.1364/OE.18.002973

[b13] Schaar J. E., Yum H. N. & Shahriar S. M. Theoretical description and design of a fast-light enhanced helium-neon ring-laser gyroscope. In *SPIE Proceedings Vol. 7949: Advances in Slow and Fast Light IV*, 794914, San Francisco, California, USA. SPIE. (10.1117/12.880786) (2011, January 22).

[b14] Deng S., Xiao Z., Zhang H., Zhao L. & Huang A. Fast-light enhanced integrated on-chip laser gyroscope for rotation sensing. *In SPIE Proceedings Vol. 8636: Advances in Slow and Fast Light IV*, 86360Q, San Francisco, California, USA. SPIE. (10.1117/12.2002946) (2013, February 02).

[b15] Qu T. *et al.* Design of a superluminal ring laser gyroscope using multilayer optical coatings with huge group delay. Sci. Rep. 4, 7098 (2014). 25403698 10.1038/srep07098 PMC4235327

[b16] Kaplan A. & Meystre P. Enhancement of the Sagnac effect due to nonlinearly induced nonreciprocity. Opt. Lett. 6, 590–592 (1981). 19710781 10.1364/ol.6.000590

[b17] Wang C. & Search C. Enhanced rotation sensing by nonlinear interactions in silicon microresonators. Opt. Lett. 39, 4376–4379 (2014). 25078181 10.1364/OL.39.004376

[b18] Srinivasan S., Moreira R., Blumenthal D. & Bowers J. Design of integrated hybrid silicon waveguide optical gyroscope. Opt. Express 22, 24988–24993 (2014). 25401532 10.1364/OE.22.024988

[b19] Hsiao H. & Winick K. Planar glass waveguide ring resonators with gain. Opt. Express 15, 17783–17797 (2007). 19551075 10.1364/oe.15.017783

[b20] Chen J. *et al.* Optimization of gyroscope properties with active coupled resonator optical waveguide structures. *In SPIE Proceedings Vol. 9378: Advances in Slow and Fast Light IV*, 93781Q, San Francisco, California, USA. SPIE. (10.1117/12.2086842) (2015, February 07).

[b21] Chen J. *et al.* Miniaturized optical gyroscope using active three-dimensional vertically coupled resonators. Optical Engineering 54, 107106 (2015).

[b22] Guillén-Torres M. A., Cretu E., Jaeger N. A. F. & Chrostowski L. Ring Resonator Optical Gyroscopes-Parameter Optimization and Robustness Analysis. J. Lightwave Technol. 30, 1802 (2012).

[b23] Terrel M., Digonnet M. J. F. & Fan S. Performance comparison of slow-light coupled-resonator optical gyroscopes. Laser & Photon. Rev. 3, 452 (2009).

[b24] Terrel M., Digonnet M. J. F. & Fan S. Performance Limitation of a Coupled Resonant Optical Waveguide Gyroscope. J. Lightwave Technol. 27, 47 (2009).

[b25] Terrel M., Digonnet M. J. F. & Fan S. Coupled resonator gyroscopes: what works and what does not. *In SPIE Proceedings Vol. 7612: Advances in Slow and Fast Light IV*, 76120B, San Francisco, California, USA. SPIE. (10.1117/12.848637) (2010, February 11).

[b26] Hu H., Ricken R. & Sohler W. Low-loss ridge waveguides on lithium niobate fabricated by local diffusion doping with titanium. Applied Physics B 98, 677–679 (2010).

[b27] Bauters J. F. *et al.* Planar waveguides with less than 0.1 dB/m propagation loss fabricated with wafer bonding. Opt. Express 19, 24090 (2011). 22109434 10.1364/OE.19.024090

[b28] Thomson D. J. *et al.* 50-Gb/s silicon optical modulator. IEEE Photon. Technol. Lett. 24, 234–236 (2012).

[b29] Soref R. A. & Bennett B. R. Electrooptical effects in silicon. IEEE J. Quantum Electronics, 23, 123–129 (1987).

[b30] Reed G. T., Mashanovich G., Gardes F. Y. & Thomson D. J. silicon optical modulators. Nature Photon. 4, 518–526 (2010).

[b31] Lee C. H., Mak P. S. & DeFonzo A. P. Optical control of millimeter-wave propagation in dielectric waveguides. IEEE J. Quantum Electron. 16, 277–288 (1980).

[b32] Pavesi L. & Vivien L. In Handbook of Silicon Photonics. Ch. 9, 439–444, (2013).

[b33] Pruessner M. W., Stievater T. H., Ferraro M. S. & Rabinovich W. S. Thermo-optic tuning and switching in SOI waveguide Fabry-Perot microcavities Opt. Express 15, 7557–7563 (2007). 19547081 10.1364/oe.15.007557

[b34] Yi Y., Shi K., Lu W. & Jian S. Phase modulation spectroscopy using an all-fiber piezoelectric transducer modulator for a resonator fiber-optic gyroscope. Appl. Opt. 34, 7383–7386 (1995). 21060612 10.1364/AO.34.007383

[b35] Espinola R. L., Tsai M. C., Yardley J. T. & Osgood R. M. Fast and low-power thermooptic switch on thin silicon-on-insulator. IEEE Photon. Technol. Lett. 15, 1366–1368 (2003).

[b36] Green W., Lee R., Derose G., Scherer A. & Yariv A. Hybrid InGaAsP-InP Mach-Zehnder Racetrack Resonator for Thermooptic Switching and Coupling Control. Opt. Express 13, 1651–1659 (2005). 19495041 10.1364/opex.13.001651

[b37] Gautam R. *et al.* Thermo-optically driven silicon microring-resonator-loaded Mach–Zehnder modulator for low-power consumption and multiple-wavelength modulation. Jpn. J. Appl. Phys. 53, 022201 (2014).

[b38] Biswajeet G., Alexander G. & Michal L. Minimizing temperature sensitivity of silicon Mach-Zehnder interferometers. Opt. Express 18, 1879–1887 (2010). 20174015 10.1364/OE.18.001879

[b39] Suzuki K., Takiguchi K. & Hotate K. Monolithically Integrated Resonator Microoptic Gyro on Silica Planar Lightwave Circuit. J. Lightwave Technol. 18, 66–72 (2000).

[b40] Corte F. G. D., Merenda M., Cocorullo G. & Rendina M. I. I. Modulation speed improvement in a Fabry–Perot thermo-optical modulator through a driving signal optimization technique. Optical Engineering 48, 705–709 (2009).

[b41] Morichetti F. *et al.* Tunable silicon CROW delay lines. *In SPIE Proceedings Vol. 7719: Silicon Photonics and Photonic Integrated Circuits II*, 771913, Brussels, Belgium. SPIE. (10.1117/12.855882) (2010, April 12).

[b42] Ye W. N., Michel J. & Kimerling L. C. Athermal high-index-contrast waveguide design. IEEE Photon. Technol. Lett. 20, 885–887 (2008).

[b43] Poon J. K. S. *et al.* Matrix analysis of microring coupled-resonator optical waveguides. Opt. Express 12, 90–103 (2004). 19471515 10.1364/opex.12.000090

[b44] Florio F., Kalantarov D. & Search C. P. Effect of Static Disorder on Sensitivity of Coupled Resonator Optical Waveguide Gyroscopes. J. Lightwave Technol. 32, 3418–3426 (2014). 10.1364/OL.39.000985 24562258

[b45] Kalantarov D. & Search C. P. Effect of resonator losses on the sensitivity of coupled resonator optical waveguide gyroscopes. Opt. Lett. 39, 985–988 (2014). 24562258 10.1364/OL.39.000985

[b46] Kalantarov D. & Search C. P. Effect of input–output coupling on the sensitivity of coupled resonator optical waveguide gyroscopes. J. Opt. Soc. Am. B 30, 377–381 (2013). 10.1364/OL.39.000985 24562258

